# Reproduction of a fossil rhinoceros from 18 mya and origin of litter size in perissodactyls

**DOI:** 10.1016/j.isci.2023.107800

**Published:** 2023-09-01

**Authors:** Xiao-Kang Lu, Tao Deng, Paul Rummy, Xiao-Ting Zheng, Yuan-Tao Zhang

**Affiliations:** 1 Department of Human Anatomy, Henan University of Chinese Medicine, Zhengzhou, Henan 450046, China; 2 State Key Laboratory of Palaeobiology and Stratigraphy, Nanjing Institute of Geology and Palaeontology, Chinese Academy of Sciences, Nanjing 210000, China; 3 Key Laboratory of Vertebrate Evolution and Human Origins, Institute of Vertebrate Paleontology and Paleoanthropology, Chinese Academy of Sciences, Beijing 100044, China; 4 University of Chinese Academy of Sciences, Beijing 100049, China; 5 Tianyu Museum of Natural History, Pingyi, Shandong 273300, China; 6 Shanwang National Geopark of China, Linqu, Shandong 262600, China

**Keywords:** Wildlife reproduction, Evolutionary biology, Evolutionary history

## Abstract

Reproductive strategy is among the most important characteristics of organism. Here, we report reproductive strategy of singleton pregnancy of a fossil rhinoceros, *Plesiaceratherium gracile*, from 18 mya of the Shanwang Basin, China. Dental and body development data revealed that after birth, the calf of *P*. *gracile* is breastfed for 2–3 years; at approximately 5 years of age, when the M2 tooth is slightly worn, the female has already reached sexual maturity and attained a size close to that of an adult and could give birth to the first calf. Furthermore, given litter size is phylogenetically conservative and closely correlates with body size, we conclude that the litter size of perissodactyls is determined by the singleton pregnancy since the Eocene. By contrast, other reproductive traits are highly variable and have a different pace of evolution, and traits observed in living rhinoceroses have been evolving at least since 18 mya.

## Highlights

• Fossil rhinoceros from 18 mya is monotocous
• Reproductive traits in living rhinoceroses have been evolving at least since 18 mya
• Litter size of perissodactyls has an origin of singleton pregnancy

## Graphical abstract

**Figure:**
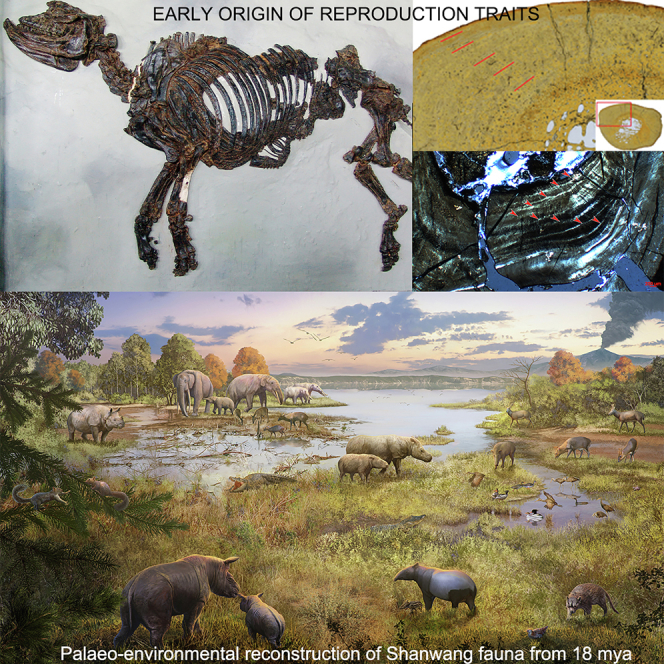

## Introduction

China has a rich fossil record of perissodactyls and other Cenozoic mammals, whose remains were traditionally regarded as ‘dragon bones’ and used in traditional medicine but have been objects of paleontological study since the end of the 19th century. Reproduction strategy decides type of species continuity and is the center of life history trade-off.^1^^,^^2^^,^^3^^,^^4^^,^^5^^,^^6^^,^^7^^,^^8^ Records show that all living perissodactyls are monotocous, but their other reproductive traits, such as gestation time and birth weight, are variable^9^^,^^10^^,^^11^^,^^12^^,^^13^ (Tables S5–S7). These indicate an interesting point the evolution of sexual reproduction. According to fossil records, from the Eocene to the Pleistocene, rhinoceroses were always dominant members of terrestrial mammalian fauna worldwide, with numerous fossils, although living rhinoceroses are represented by only five species.^14^^,^^15^^,^^16^^,^^17^^,^^18^^,^^19^ However, there are no publications on fossil rhinoceroses in terms of reproduction traits, and the origin of such traits and the reason for their differences remain unknown. Therefore, we aimed to bring certain fossil species into the discussion and provide valuable data for understanding reproductive evolution in rhinos.

This study is based on a well-preserved skeleton of pregnant female rhino with an unborn fetus, several immature skeletons and skulls, and the microstructure of teeth and limb bones to infer the reproductive traits of fossil rhinoceroses based on tooth eruption and body growth rate data. These materials of *Plesiaceratherium gracile* Young, 1937 were produced in the Early Miocene (18 mya) in the Shanwang Basin, a famous locality with diatomaceous shales and well-preserved fossils.^20^^,^^21^

## Results

### Dental development stage

**Figure 1:**
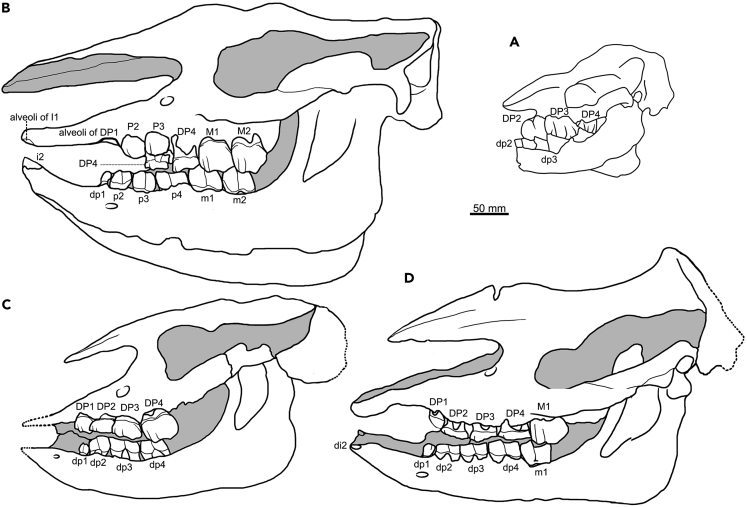
Immature skulls of *Plesiaceratherium gracile* from the Early Miocene of China (A) Fetus, GSP 125; (B) subadult, with the second molars slightly worn, S700017; (C) juvenile, with all deciduous teeth erupted, S700016; (D) juvenile, with the first molars slightly worn, STM 44–64.

Observation of eight new juvenile skulls (STM 44–77/113/116, S700016, and GSP 126) and skeletons (STM 44–64, S700017, and GSP 125) suggested the following dental eruption sequences: DP2, DP3, DP4, DP1, M1, M2, P2, P3, P4, and M3 (Figure 1; Figures S1–S5). There were no visible differences in the crown height and eruption level between P2 and P3; both may have erupted simultaneously.

**Figure 2:**
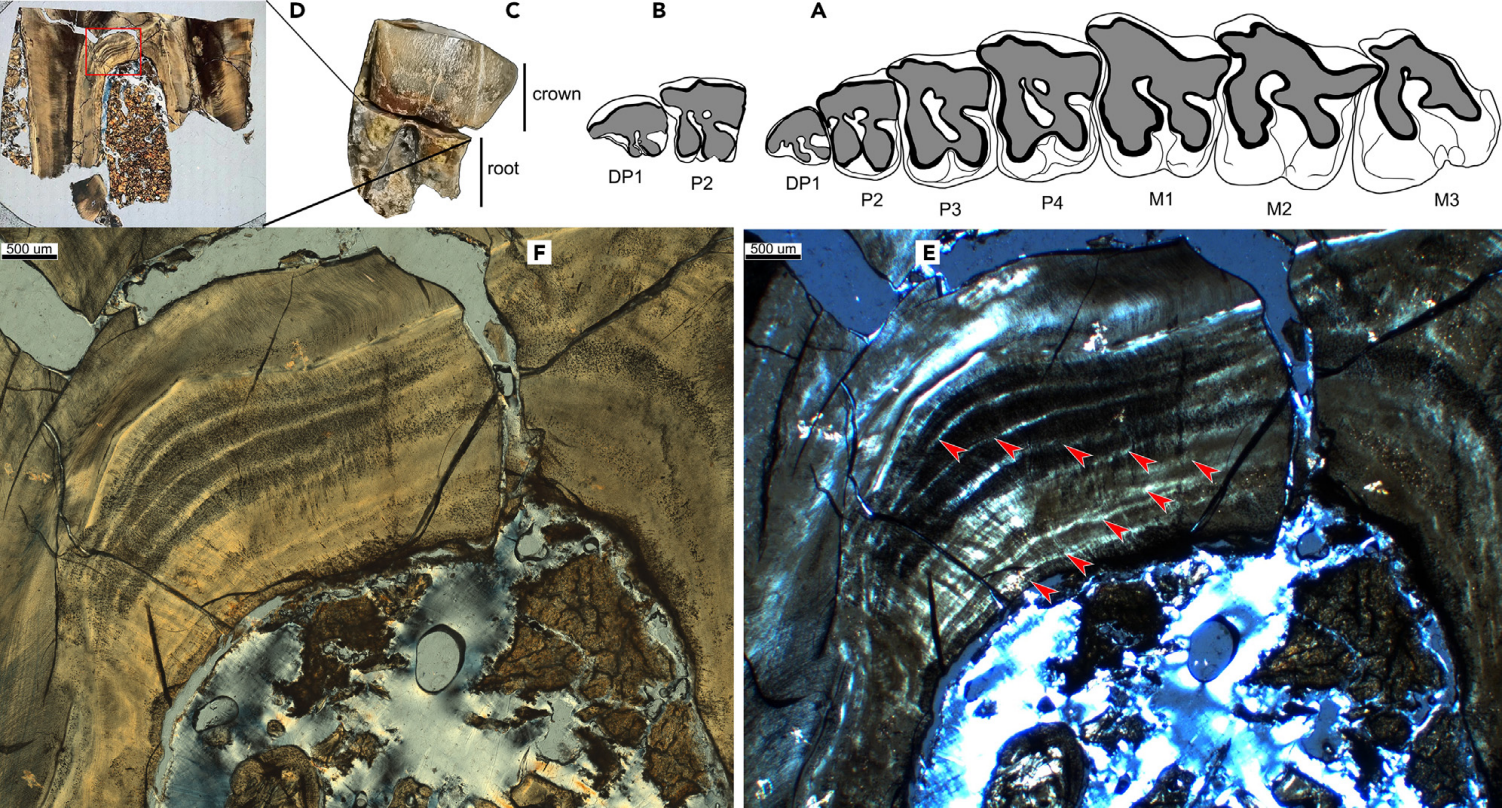
Upper cheek teeth and section of DP1 of *Plesiaceratherium gracile* from the Early Miocene of China (A) Occlusal view of upper cheek tooth row of adult individual STM 44–67; (B) occlusal view of the isolated upper cheek teeth DP1–P2 (GSP 127); (C) DP1, labial view, showing crown and root, both have been detached for preparing of section; (D) antero-posterior section of root, overview; (E and F) cement at the interradicular area, red arrowheads indicate the annual incremental lines under polarized light.

The new maxillary fragments of DP1 and P2 (GSP 127) indicated tooth wear similar to that of new adult skulls (STM 44–67), which crossed the threshold of body maturity, with the M3 being slightly worn. These results indicated the same dental development stage in the two samples. In the cross-section of DP1 (GSP 127), nine or ten annual incremental lines were observed in the interradicular region of the cementum (Figure 2). The estimated age of GSP 127 and STM 44–67 was 10–11 years.

The dental development of *P. gracile* matched well with records of living rhinoceroses based on known age samples^22^^,^^23^ (white rhinoceros *Ceratotherium simum*, specimens N20, 27, and K1), eruption sequences, and time schedules. As eruption and wear of the third molar are considered signs of body maturity, we suggest that the body maturity of *P. gracile* takes at least 10 years.

### Body growth rate

**Figure 3:**
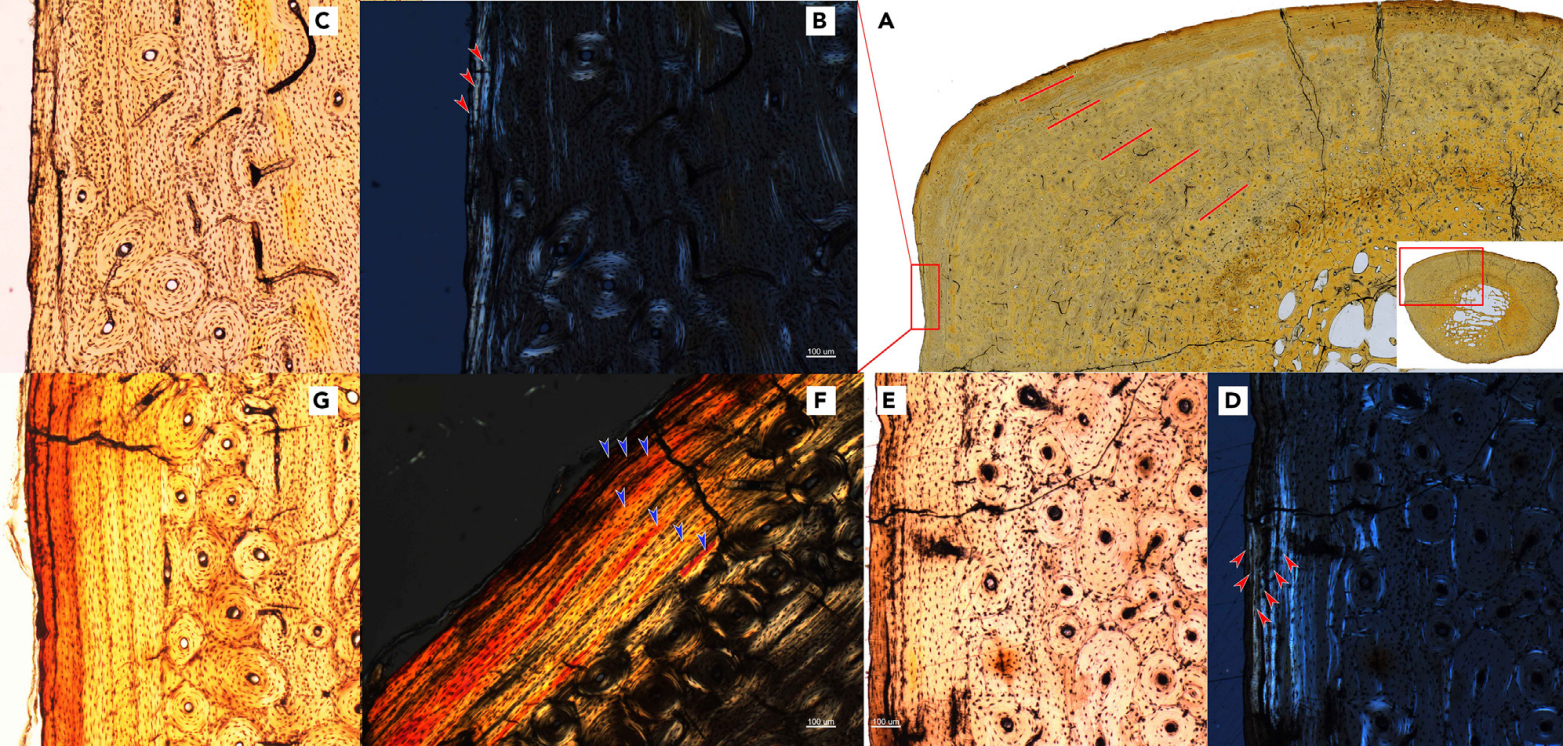
Cross section of limb bones of individual adult STM 44–167 of *Plesiaceratherium gracile* from the Early Miocene of China (A) Dorsal-lateral area of Mt IV, red lines indicate five annual circle growth lines; (B and C) external area of a section of Mt IV, red arrowheads indicate three external fundamental lines under polarized light; (D and E), external area of a section of the radius, red arrowheads indicate six external fundamental lines under polarized light; (F and G) blue arrowhead indicates seven external fundamental lines under polarized light.

Measurements of the new materials showed a growth rate corresponding to the tooth development stages (Figure 3; Tables S1 and S2). In the fetus of the pregnant rhinoceros (GSP 125), the deciduous teeth DP2 and DP3 were above the bone level of the alveolus, while DP4 was below. Its skull length was 221 mm, approximately 34% of the average adult length. All deciduous premolars were worn in the skeleton S700016, and the basal length of the skull increased to 66% of the average adult length. The skull length grew to approximately 80% of the average adult length when M1 was slightly worn (STM 44–64) and up to 90% when M2 was slightly worn (S700017).

In STM 44–64, in which all deciduous cheek teeth erupted, the proximal and distal extremities of the long bone were detached from the shaft. For the slightly worn M2 of S700017, the proximal and distal extremities were fused to the shaft. In STM 44–64, the humerus grew to 71% of the mean adult length, radius 76%, third metacarpus 83%, femur 65%, tibia 74%, and third metatarsal 81%. In S700017, these long bones grew to > 90% of the mean adult length, and the third metatarsal grew to 97%.

**Figure 4:**
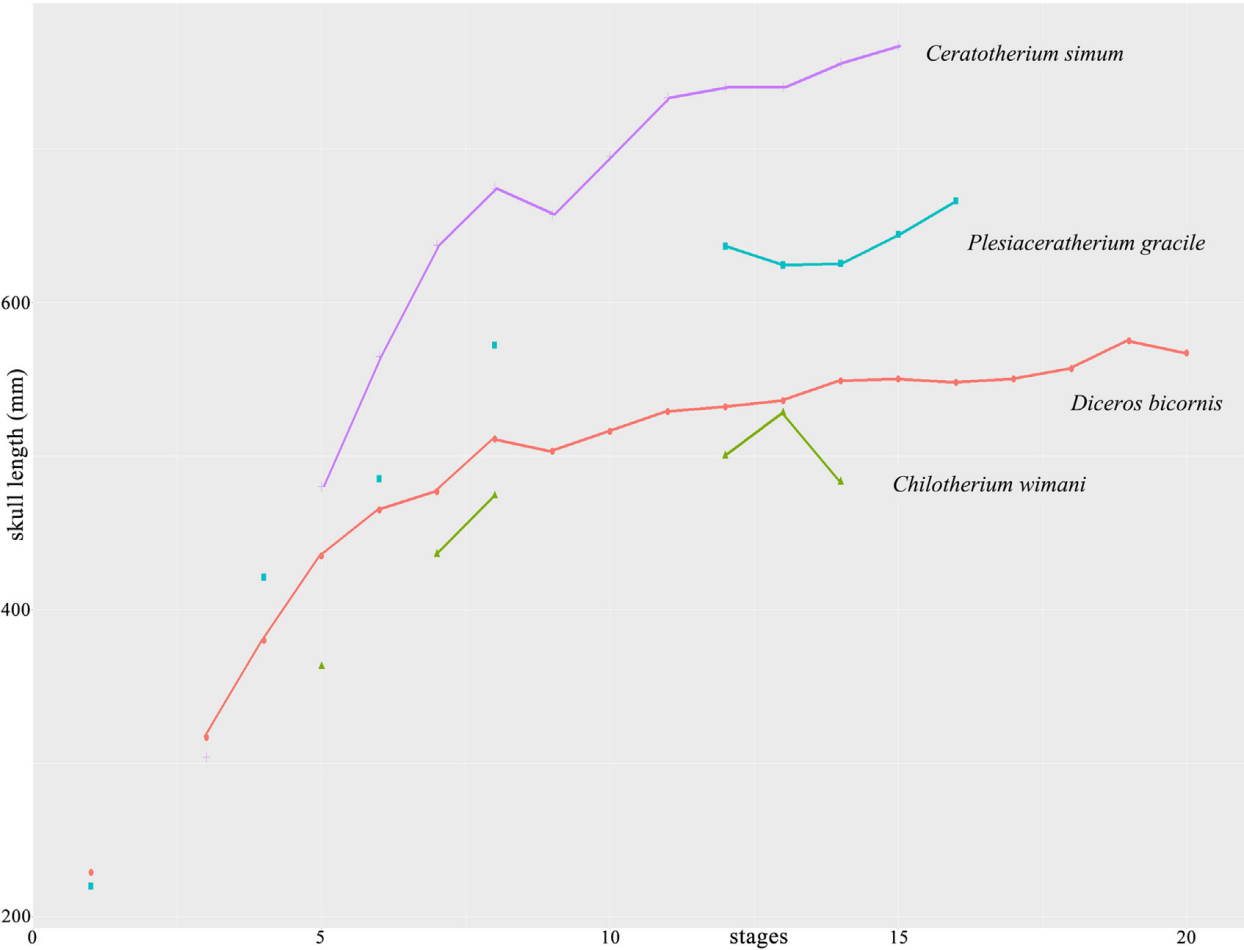
Length plot of skull, data from two living and two fossil rhinoceroses with development, showing both rapid and plateau periods of growth Measures of living rhinoceroses are from full development series, while records of fossil rhinoceros are relatively limited. Length data of living rhinoceroses are presenting as mean value, but fossils species are not. See more data in Table S8.

To determine the growth rate more accurately with age, we sectioned several limb bones from a single skeleton (STM 44–167), which did not have the skull bone, and the developmental stage was unknown (Figure S6). Two proximal long bones, the radius and tibia, and a distal long bone, Mt IV, have the annual growth line. The internal part of Mt IV had five annual growth lines. For growth lines of the external fundamental system, 5–6 growth lines were observed in sections of the radius and the tibia, but only 4 growth lines were observed in the Mt IV because its external edge is not completely preserved (Figure 3). This indicates a faster growth period in the first five years of life. This age schedule for body growth was demonstrated using the fossil skeleton S700017. As mentioned previously, the body size of this skeleton (S700017) was close to that of an adult and was in a plateau period of growth (Figure 4). Considering the dental eruption stage (the second molar was fully erupted and slightly worn), the age of skeleton S700017 was probably 4–6 years old. This result is roughly consistent with the estimated age of maturity using equation of body size to life history traits, as well as living rhinoceroses (Tables S1–S4).

## Discussion

### Lactation period

In this study, a well-preserved skeleton of pregnant female (GSP 125) confirmed the reproductive strategy of singleton pregnancies in *Plesiaceratherium gracile* 18 mya. Under this strategy, females would have to take the risk of no offspring within a breeding season, and neonatal survival relies on maternal care to a great extent, with adaption to lactation, protection, and others.^4^^,^^24^^,^^25^^,^^26^ As weaning indicates the end of lactation and the independence of nutrition for an infant, it is closely related to tooth eruption and body growth. Generally, it occurs with considerable body growth and the eruption of the first molar.^5^^,^^27^^,^^28^

The new juvenile skull, S700016, with all deciduous teeth erupted and worn, had a basal skull length of 66% of the average adult length. When the first molar was slightly worn on STM 44–64, the skull length was 80% of the average adult length. Although weaning weight data were inaccessible for *P. gracile*, its growth rate was comparable to that of live rhinoceroses. With the full complement and wear of deciduous teeth, at approximately 1 year of age, the basal skull length of living rhinoceroses grows to > 60% of the average adult length, and during the eruption of M1 at 1–3.5 years old, the skull length could grow to 84%.^11^^,^^12^^,^^22^^,^^29^^,^^30^

Regardless of the difference in adult body weight, the five living rhinoceroses share a similar growth rate, at least in their childhood, and their calves weigh approximately 400 kg at 12 months of age in captivity.^31^^,^^32^ In the wild, the weaning event of living rhinoceros is delayed up to 2–3 years old, later than the 1–2 years old in captivity.^33^^,^^34^^,^^35^^,^^36^ Given the similarities between *P. gracile* and existing rhinoceroses in terms of dental eruption time and body growth rate, we confirmed that STM 44–64 was weaned. Similar to those of living rhinoceroses, the calves of *P. gracile* were probably weaned at 2–3 years of age. At this age, all deciduous cheek teeth erupted, M1 erupted, and the body reached a considerable size (approximately 80%).

### Age of sexual maturity and first birth

To estimate the reproduction of fossil species, one must first confirm the reproductive age, which indicates sexual maturity and first conception. The age of living rhinoceroses is easily determined by several signals, such as oestrus cycle activity, mating behavior, and the presence of a calf accompanied by its mother.^33^^,^^34^^,^^37^ However, in the present study, the only accessible data for inferring the reproduction age of *P. gracile* were those for body development.

Living female rhinoceroses generally give birth to their first calf at 4–5 years of age.^9^^,^^13^^,^^23^^,^^33^ In the range of 4–9 years, M2 was slightly worn, and the skull lengths of *Diceros bicornis* (Linnaeus, 1758) and *Ceratotherium simum* (Burchell, 1817) were 86–93% and 90–96% of the average adult length, respectively.^9^^,^^13^^,^^22^^,^^23^ This indicates that the body size grew to a considerable degree, such that the female was able to give birth.

The subadult individual (S70017) of *P. gracile* with a slightly worn M2 grew to 89–95% of the average adult size (from measurements of the skull and postcranial bones). This growth rate was comparable to that of living rhinoceroses.^9^^,^^13^^,^^22^^,^^23^ Additionally, 9–10 annual growth marks were counted in the cross-section of Mt IV (STM 44–167), including five internal annual growth lines and four to five lines in the external fundamental system, implying a rapid growth period of limb bones within the first five years of life (Figures 3 and 4). It is clear that at the beginning of the wear of the second molar, at approximately 5 years of age, the female *P. gracile* had already reached sexual maturity, and its body could house a fetus and give birth to the first calf.

### Evolution of reproduction

The reproduction of mammals has long been discussed by many studies, and its relationship with body size and phylogeny is among the most interesting topics.^2^^,^^4^^,^^5^^,^^6^^,^^27^ This study on *Plesiaceratherium* from the Early Miocene of revealed a reproduction strategy of singleton pregnancy similar to that of living rhinoceroses, including traits of small litter size, long lactation period, delayed age of maturity, and small lifetime offspring number.

The number of offspring per pregnancy depends mainly on genetic factors.^38^^,^^39^ In several different populations of monotocous sheep, genetic regulation could affect the ovulation rate and litter size.^40^^,^^41^ At a certain taxonomic level, data of living mammals showed that litter size is phylogenetically conserved; for example, all taxa of Perissodactyla, as well as those of Primates, Cetacea, and Proboscidea, are monotocous; however, Rodentia is polytocous.^42^^,^^43^ Conversely, within Artiodactyla, which comprises the most successful living large mammals, litter size is variable: Suidae is polytocous, while Tragulidae is monotocous.

Phylogenetically, *Plesiaceratherium* studied herein and all five living rhinoceroses are members of the family Rhinocerotidae. The earliest record of the family Rhinocerotidae, represented by the genus *Teletaceras*, is from the Middle Eocene (45 mya),^44^ slightly later than that of the early basal genus (*Hyrachyus*) of the superfamily Rhinocerotoidea from the late Early Eocene (50 mya).^45^ It is easy to understand the similarities in reproduction between *Plesiaceratherium* and the five living rhinoceroses because they are not only phylogenetically close but also have a similar body size, because of size is closely related with litter size.^46^ However, *Teletaceras* and *Hyrachyus* are small in size, with a skull length of 200–350 mm, close to size of the fetus of *Plesiaceratherium*.^44^^,^^45^ No report has discussed whether the smaller and primitive rhinoceroses are similar to their larger descendants in terms of the reproduction strategy of singleton pregnancy.

In fact, the other fossil record of litter size in Perissodactyla is from a smaller and older equid *Eurohippus* from the Middle Eocene (47 mya) in the locality of Grube Messel in Germany.^47^ Its pregnant skeleton provides an undoubtful evidence not only for the early origin of monotocous mammals, but also for the existence of small-sized mammals, with body length of approximately 600 mm and body weight of approximately 10 kg, much smaller than the fetus of *Plesiaceratherium*.^47^ Moreover, similar to its larger relative *Bos*, the smallest living ungulate *Tragulus*, with a body weight of 2 kg and with the oldest ancestor from the Middle Eocene, is also monotocous.^48^^,^^49^

**Figure 5:**
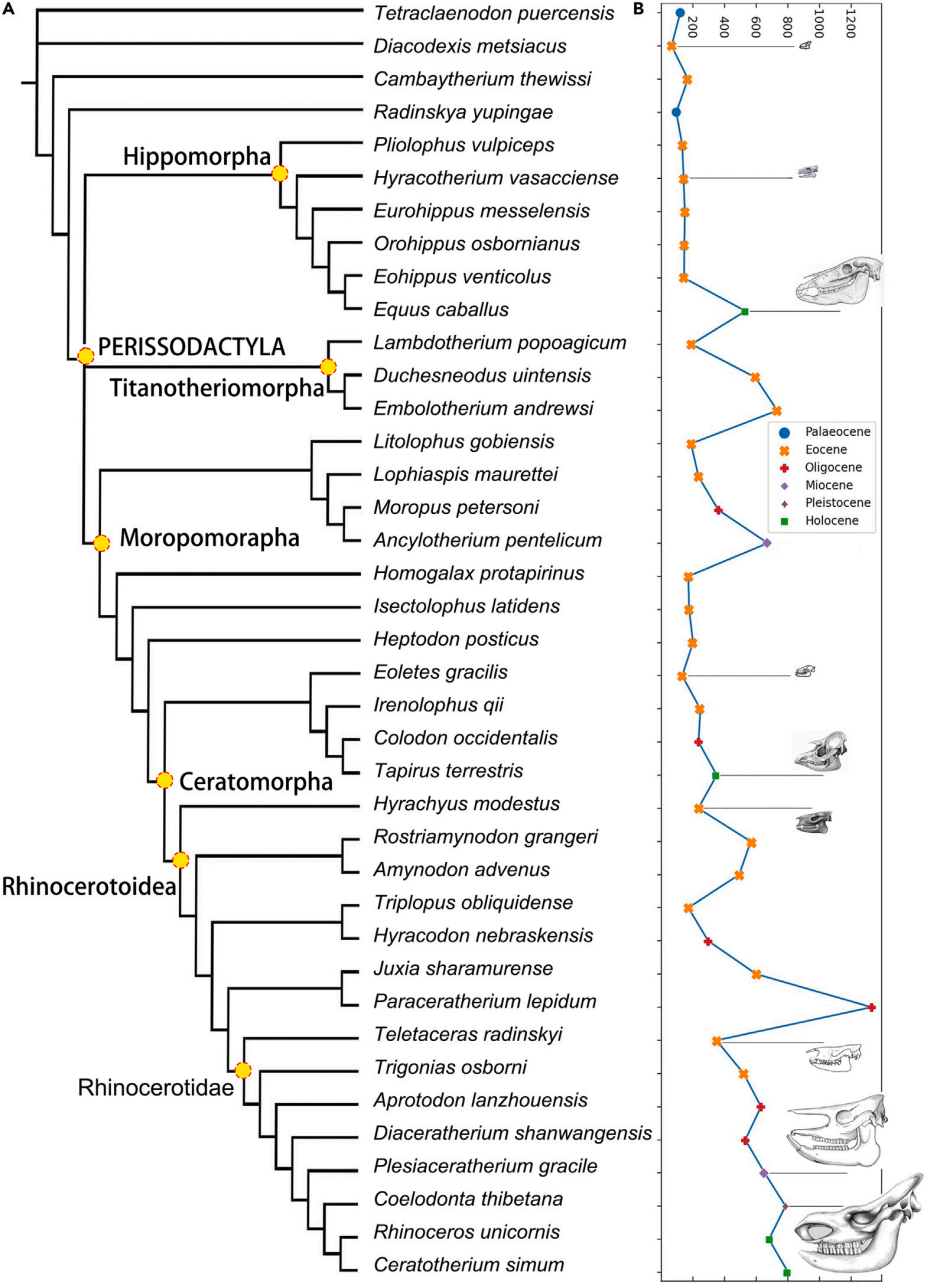
Size of perissodactyls through the Paleogene to the Quaternary, phylogenetic relations adopted previous works (A) Phylogeny relationship of perissodactyls, this topology was generated by combining previously published trees; (B) line chart showing size difference among taxa from different geological age, and skull drawings shared the scale. See more details about size in Table S9.

The fossil records from the first million years after the Cretaceous-Paleogene mass extinction demonstrated a fast increasement of maximum mammalian body mass.^50^ Soon afterward, perissodactyls were prosperous and composed of several stem-based clades, such as Isectolophidae, Brontotheriidae, and Palaeotheriidae, as well as crown-based clades, such as Equidae, Rhinocerotidae, and Tapiridae, and even Anthracobunia, which was recently untied into Perissodactylamorpha.^14^^,^^51^^,^^52^ *Cambaytherium* from the Early Eocene is the most common genus of Anthracobunia, and its minimum body weight is approximately 10 kg, close to that of other contemporary relatives, and its maximum weight is up to 99 kg.^52^^,^^53^ The body size of other basal perissodactyls, such as *Lambdotherium*, *Litolophus*, *Homogalax*, and *Triplopus*, is relatively larger (Figure 5) (Table S9). The evolution of body size in fossil horses is frequently depicted as a gradual, progressive trend toward increased body size.^54^^,^^55^^,^^56^ The widely accepted view is that living equids originated from North America (including *Hyracotherium*, *Pliolophus*, *Arenahippus*, and others, which have a body weight of 10–25 kg), while their contemporary relatives originated from Europe, including *Eurohippus*.^51^^,^^53^

The maximum offspring number is the center of reproduction trade-off, and its relationship with body size has long been discussed.^4^^,^^57^ According to the equation of body weight against litter size, mammals with a body weight greater than 1 kg have decreased litter size.^46^ To date, all unquestionable stem fossil perissodactyls have a body weight greater than 10 kg, and living perissodactyl weigh 150–3000 kg (Table S9). Based on the pregnant skeleton of *Eurohippus* with a single fetus, we postulated that the evolution of litter size in Perissodactyla is determined by singleton pregnancy since the Eocene, which is a very special reproduction strategy due to the high risk of no offspring at each breeding season. To employ this strategy, small-sized mammals adopt a method different from that of their larger relatives. Using equations against body size,^58^^,^^59^ the primitive rhinoceros *Teletaceras* weighs 29 kg, has a lifespan of approximately 20–24 years, has an age of maturity of approximately 2 years, and is much shorter and younger than living rhinoceroses. Even among the reproduction traits of living rhinoceroses, there are visible differences in the gestation length and consequent birth weight of calves, especially between *Ceratotherium* and *Dicerorhinus*. Among the three living clades of perissodactyls, the differences in reproduction traits are more visible.^42^^,^^43^ To trade off the high mortality of adult individuals and singleton pregnancy, small-sized ungulates tend to produce offspring as early as possible.^4^ Generally, other reproduction traits of living rhinoceroses, including gestation length, birth weight, and age of maturity, are variable and evolve at a pace different from that of litter size, which is phylogenetically conserved.

### Limitations of the study

The oldest perissodactyls are from the Early Eocene, but the genomic timescale suggested that the perissodactyl clade diverged since the Palaeocene.^60^ It remains an open question whether genotype could influence phenotype, which has taxonomic significance in Perissodactyla. Reproduction information of fossil mammals is very scarce. In addition, so far, no fossil from the Palaeocene has been classified as a perissodactyl. Among the oldest and smallest fossil ungulates, *Diacodexis* and *Radinskya* from the Early Eocene (53 mya) have a size comparable to that of rodents (less than 1 kg), such as the living *Sylvilagus* or *Sciurus*, but smaller than that of the living smallest artiodactyl *Tragulus*.^61^^,^^62^^,^^63^^,^^64^ The correlation of reproduction with the life history of fossil species remains a barren field, with very limited publications. Studying the litter size of such small paleo-mammals will provide more evidence for reproductive evolution.

## STAR★Methods

### Resource availability

#### Lead contact

Further information and requests for resources and reagents should be directed to and will be fulfilled by the lead contact, Xiaokang Lu (luxiaokang@ivpp.ac.cn).

#### Materials availability

This study introduces many valuable fossils for measures and make thin section, those are from the Early Miocene Shanwang Basin and taxonomically are referred to *Plesiaceratherium gracile*, and housed in two museums with detectable numbers, namely Tianyu Museum of Natural History (STM), Linqu County Museum (S), and Shanwang National Geopark of China (GSP), China. If there are any need to take a direct observation on these materials, please feel free to contact the lead contact.

To reconstruct the development stage, a total of eight unpublished juvenal skeletons and skulls of *P. gracile* with complete teeth row are included in this study (skull, STM 44–77/113/116, S700016, and GSP 126; skeletons, STM 44–64, S700017, and GSP 125). All specimens are accessible for the shape information, but the measures are limited to two skeletons and one skull due to others are sealed in glass box, like the situation we have mentioned (Lu et al., 2021). While there are biochemical markers that have been used in the past to estimate weaning in fossil teeth, we did not have access to those assays and have instead relied on histological markers. One specimen of maxillary fragment (GSP 127, with DP1 and P2) and one adult skull (STM 44–67) are used to estimated accurate age by counting the annual record of cementum. Several limb bones of one skeleton (STM 44–137) are sectioned for reconstructed of growth rate based on the annual growth marks.

### Experimental model and subject details

This paper did not use any experimental animals.

### Method details

#### Teeth eruption and attrition

In following section, we compare and match each new materials to the development stages of living rhinoceros that are established based on dental development and annual records of cementum.^22^^,^^23^^,^^29^ The wear stages used in this study, such as ‘slightly wear’ and ‘moderately wear’, follow the class criteria suggested by Hillman-Smith et al.^29^

The alveolar bone is clearly and fully exposed on all new specimens, and it is easy to recognized whether tooth is above the bone level. Given the ectoloph of cheek teeth of rhinoceros will inevitably change the outline and became shallow W-shape in lateral view once reaching the slightly wear stage, we would use this as a reference of early stage of attrition. In addition, with growth, the crown part will be further worn and the root will be exposed more and more, these would be used to judge whether tooth is fully erupted and to compare the wear stage. Due to variations in individual eruption and attrition of dentition, it is not necessarily to refer specimens to a particular age but to an age range, as previous studies did.

#### Counting annual lines of cementum

In order to get accurate age of development stages of *P. gracile*, isolated teeth are prepared for histology section. DP1 and P2 from one maxillary fragment (GSP 127), with development stage similar to one complete skull (STM 44–67). On one hand, the development stage will be assigned to both specimens based on their attrition degree. On the other hand, accurate age will be gained by counting the annual records of cementum DP1.

In living African rhinoceros, DP1/dp1 are erupted within one year old, to be the last pair erupted deciduous teeth. Our primary observation on new fetus of *P. gracile*, which is ready to be given birth, crown of dp1 is forming although later than other deciduous teeth. Phylogenetically, dental eruption schedule is not earlier in advanced species than in primtive’s. We therefore could confirm that DP1 of *P. gracile* would be erupted within one year old, and counting of its incremental lines are roughly similar to its real age with error range less than one year.

#### Body growth

For estimation of growth rate and skeletal mature time, we measure size of skulls, mandibles and postcranial bones from several development stages, investigate the annual growth marks on histology section of long bones, and correlated results of these two aspects with the roughly assigned age based on teeth eruption and attrition.

Due to some materials are sealed in the glass box, only two skulls and three skeletons are measured. We follow measurements protocols established by Guerin^18^ for both skull, mandible, and limb bones, but with a revision: ‘distance between M3 and occipital condyle’ is revised as ‘distance between last tooth and occipital condyle’.

Six postcranial bones from one single skeleton of *P. gracile* (STM 44–167), which is an adult individual based on size measures of limb bones although the skull is lost, are used for testing histology section, including a left tenth rib, left radius, right second metacarpus (Mc II), right third metacarpus (Mc III), left tibia, right fourth metatarsus (Mt IV). The middle part of shaft was sampled for each long bones, but the sample of rib belongs to the distal part of the tenth rib. Due to the shaft of tibia has been crushed, the anterior-medial angle was lost in the course of preparing the section. A piece of anterior side of the radius has been lost too.

To obtain the data of growth, samples of bone blocks were embedded in epoxy resin Araldite 2020, then cut with an STX-202A diamond wire cutting machine. We get a thin bone block with thickness broadly 500 μm. The surface that will not be tested is polished using P320 to P4000 abrasive paper and mounted to a glass slide using the same epoxy resin. The surface prepared for testing is polished to 100–150 μm, using abrasive paper. Finally, the slides will be covered with a DPX medium. Sections are tested using the optical microscope with option of polarized light of Olympus corporation (BX51-P), and the panorama picture of each section is taken using camera of Canon company (700D) and microscope of Zeiss company (Axio Lab. A1). All cementum lines are easy to recognized, and bright lines are counted.

#### Reproduction traits

The final step of this study is aimed to reconstruct reproduction for this extinct fossil rhinoceros as complete as possible. The pregnant female told us that the offspring number of each birth is single one. Several reproduction-related variables of life-history are easily recognized that have been pointed out in above sections, including the dental eruption times, body growth rate, and longevity. We have to refer to the life history of extant rhinoceros, and make a postulation on these traits. For longevity and age of maturity, we used three established equations, compare results, and discuss reliability according the records of extant rhinoceros, these are listed in Suppl. tables. In addition to these, other life history variables are not easily recognized for fossil taxa, including the gestation length, age at weaning, age at first reproduction, interbirth interval, and number of offspring. Some postulation will be strong, such as weaning time that correlates best with emergence of M1, but some is weak, such as number of offspring.

### Quantification and statistical analysis

This paper has no quantification analysis.

In this paper, we provided two scatter diagrams, namely Figures 4 and 5, using the statistical software R, by the package “ggplot2” and without converting parametric objects. In addition to this, there has no other statistical analysis.

## Data Availability

• This paper did not report original code. • This paper did not include any data need to be deposited in the public website. • All datasets used in this paper are provided as supplemental information. Any additional data, including the thin section, required to re-analyse the data reported in this paper is available from the lead contact upon request.
