# Supplementary material for: Reproduction of a fossil rhinoceros from 18 mya and origin of litter size in perissodactyls
Source: iScience. 2023 Sep 1;26(10):107800. doi: 10.1016/j.isci.2023.107800 (PMC10514446; doi:10.1016/j.isci.2023.107800)
Supplement: Document S1. Figures S1–S7 and Tables S1–S9 [file mmc1.pdf]

iScience, Volume *26*

Supplemental information

Reproduction of a fossil rhinoceros from 18 mya and origin of litter size in perissodactyls

Xiao-Kang Lu, Tao Deng, Paul Rummy, Xiao-Ting Zheng, and Yuan-Tao Zhang

Supplementary Information

Supplementary figures

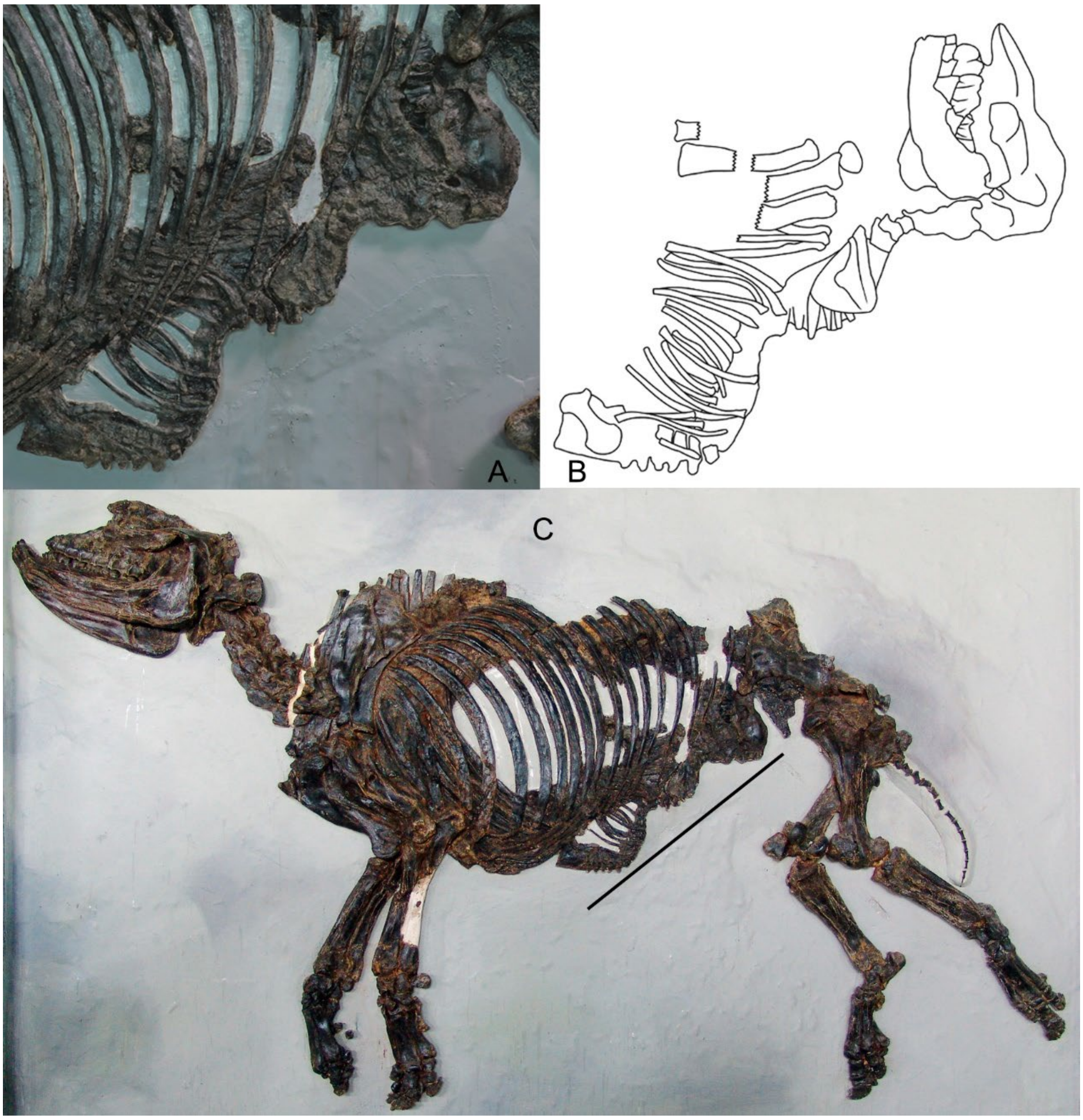

Figure S1. Pregnant skeleton of *Plesiaceratherium gracile* from the Early Miocene 18 mya. a, b, foetus fossil and drawing; c, adult skeleton, with third molar at moderately worn stage. Related to Figure 1.

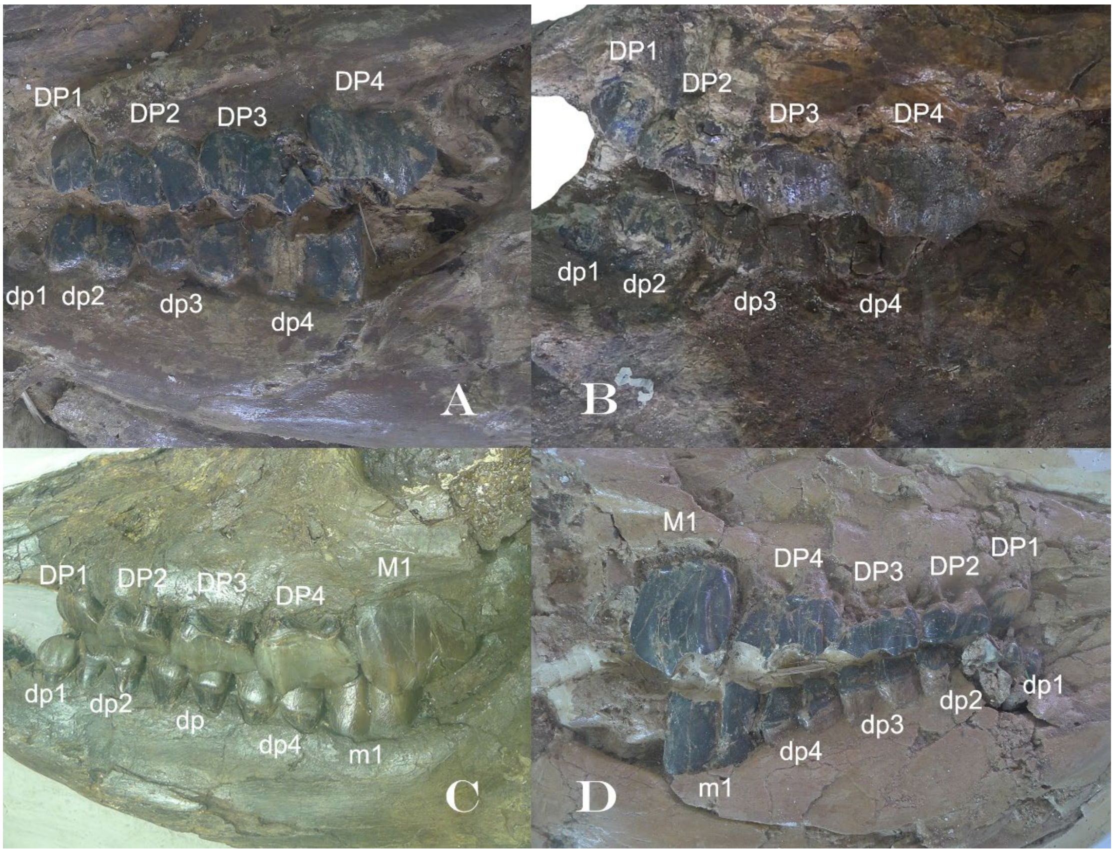

Figure S2. Skulls of *Plesiaceratherium gracile* with tooth eruption sequence, A, STM 44-116; B, STM 44-113; C; GSP 126; D, STM 44-77. All samples are sealed in glass box, not to scale. Related to Figure 1.

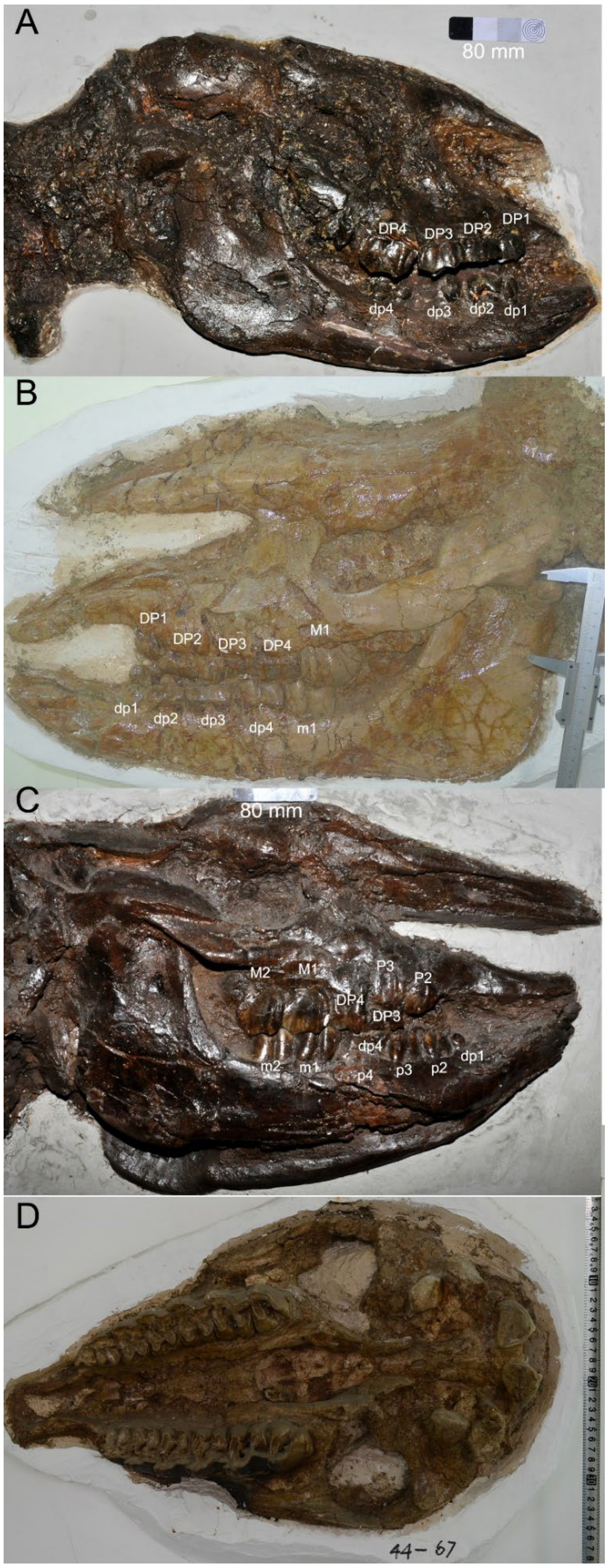

Figure S3. Skulls of *Plesiaceratherium gracile* with tooth eruption sequence, A, S700016; B, STM 44-64; C, S700017; D, STM 44-67. Related to Figure 1.

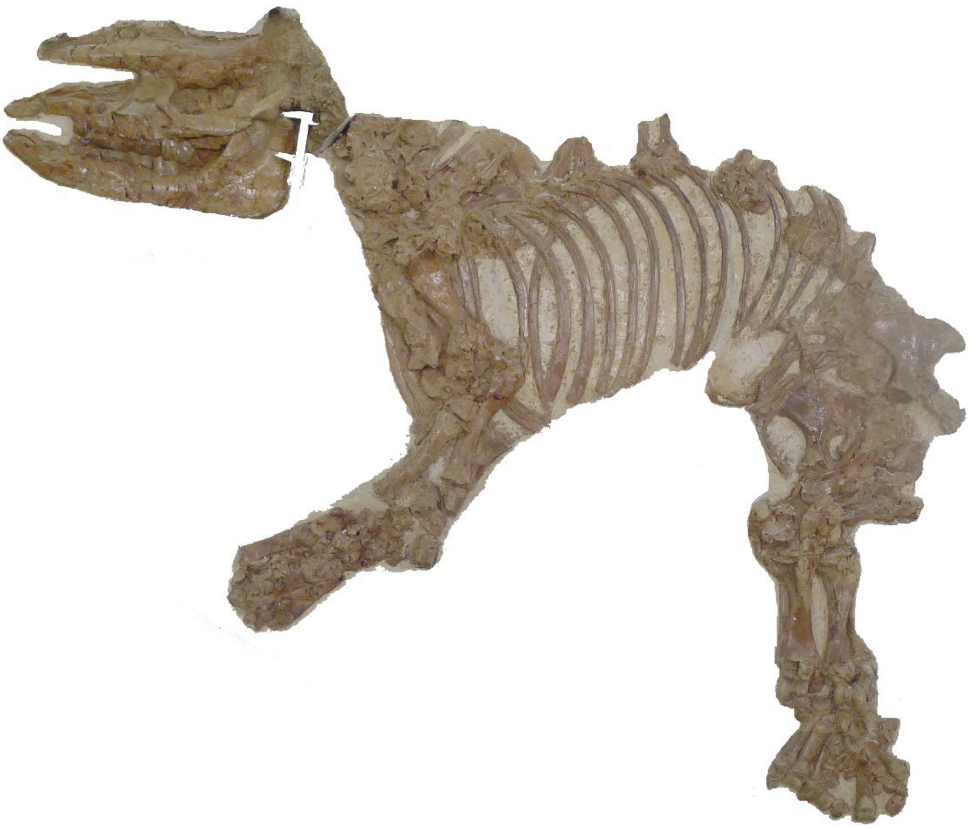

Figure S4. Skeleton of *Plesiaceratherium gracile*, STM 44-64. Related to Figure 1.

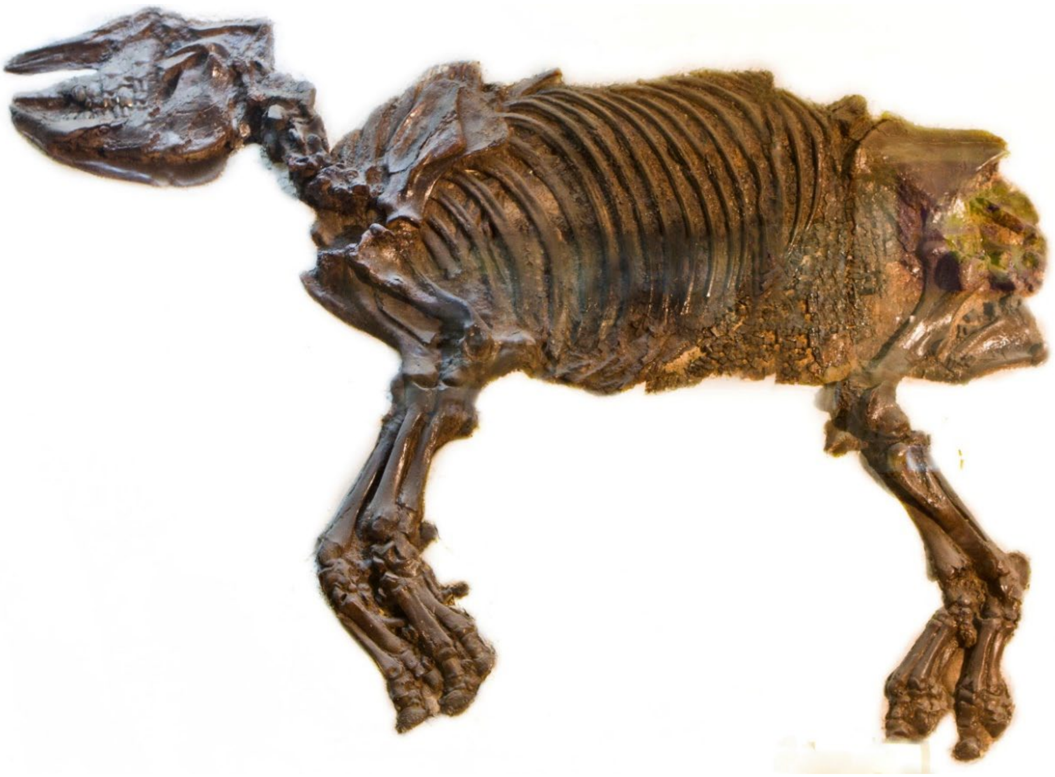

Figure S5. Skeleton of *Plesiaceratherium gracile*, S 700017. Related to Figure 1.

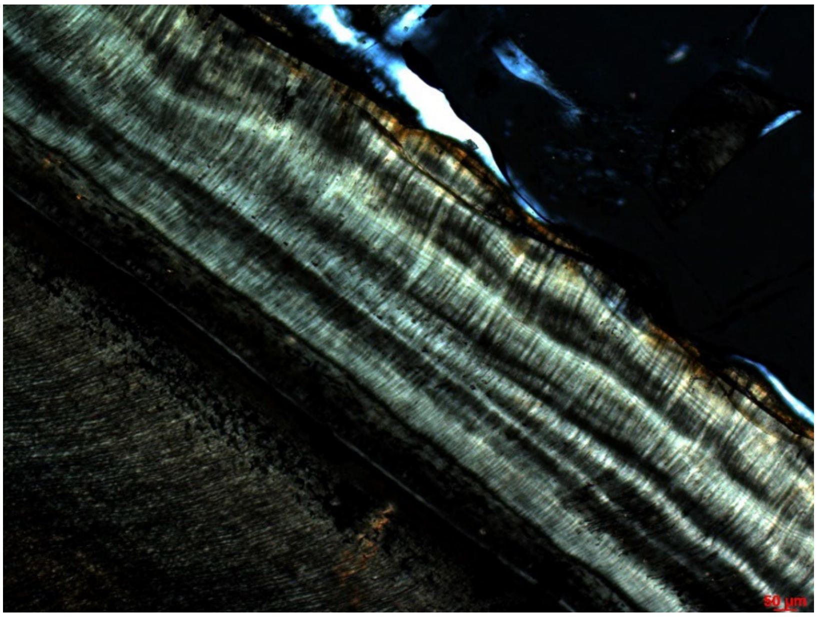

Figure S6. Cementum around the root below the crown-root joint of DP1 (GSP 127) of *Plesiaceratherium gracile*, the annual growth lines are badly stratified. Related to Figure 2.

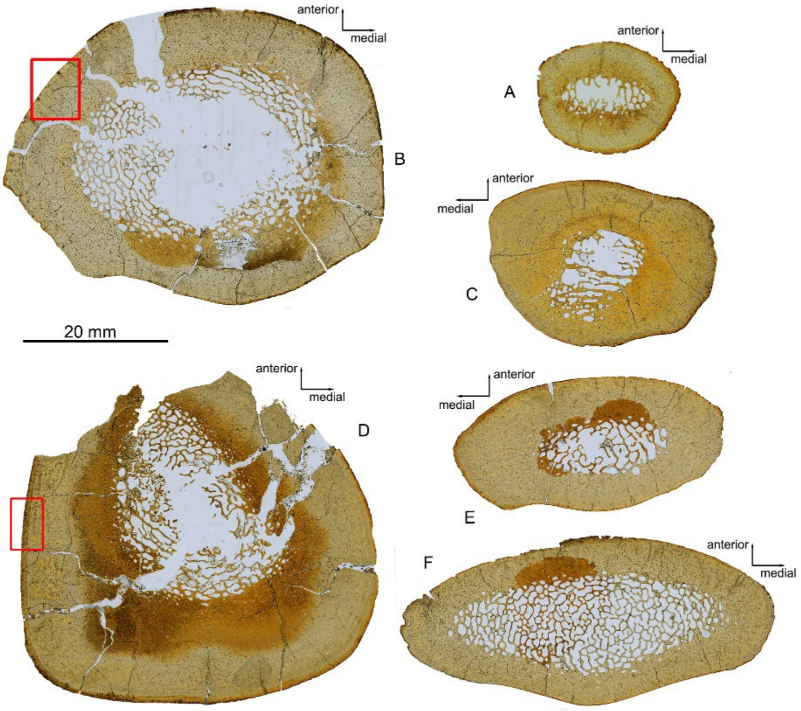

Figure S7. Cross-section of limb bones of skeleton (STM 44-167) *Plesiaceratherium gracile*. A, rib; B, radius; C, Mt IV; D, tibia; E, Mc II; F, Mc III. The red box of radius and tibia marked the position of exaggerated pictures in text figure 3. Related to Figure 3.

Supplementary tables

Table S1. Measures and ratio of skull and mandible of *Plesiaceratherium gracile* from the Early Miocene Shanwang Basin, China (mm). Related to Figure 1 and 4.

	1	2	3	4	5	6	7	8	9	10	11	12	13	14	15	16	17	18
measures	421	364	348	98	157	193	210	187	53	138	41	159	341	74	153	94	64	140
ratio	0.66	0.57	0.54	0.41	0.56	0.62	0.62	0.68	0.7	0.42	0.34	0.62	0.62	0.54	0.64	0.61	0.67	0.57
measures	510	490	432	161	148	178	204	234	46	198	80	190	457	118	188	124		
ratio	0.8	0.76	0.66	0.67	0.53	0.57	0.6	0.85	0.61	0.6	0.66	0.74	0.83	0.86	0.79	0.8	0	0
measures	572	587	593	236	245	276	308	239	56	294	72	216	480	132	218	124	78	203
ratio	0.9	0.92	0.91	0.99	0.87	0.88	0.9	0.87	0.74	0.89	0.59	0.84	0.88	0.96	0.91	0.8	0.81	0.83

1. Occipital condyle-Premaxillae; 2. Occipital condyle-Premaxillae; 3. Occipital crest-Nasal bone; 4. Nasal length; 5. Occipital condyle-Postorbital process; 6. Occipital condyle-Supraorbital process; 7. Occipital condyle-Anteriorbital process; 8. Occipital condyle-M3; 9. Nasal notch-Orbit; 10. Nasal tip-Orbit; 11. Maxillary diastema; 12. Upper cheek teeth row; 13. Mandible length; 14. Mandible symphysis; 15. Mandible condyle height; 16. Ramus width; 17. Mandible diastema; 18. Lower cheek teeth row. Ratio is to mean adult lengths that are from Lu et al. (2020).

.

Table S2. Measures and ratio of postcranial bones of *Plesiaceratherium gracile* from the Early Miocene Shanwang Basin, China (mm). Related to Figure 1 and 4.

Specimens		Scapula Length	Humerus Length	Radius Length	Mc II Length	Mc III Length	Mc IV Length	Femur Length	Tibia Length	Mt II Length	Mt III Length	Mt IV Length
44-64	measures	226	275	268		158		310	260	130	145	126
	ratio to mean adult length	0.5622	0.7143	0.7614		0.8272	0.9359	0.654	0.7386	0.828	0.8146	0.8235
S700017	measures	355	355	317	158	178	146	422	325	165	174	145
	ratio to mean adult length	0.8831	0.9221	0.9006	0.9576	0.9319		0.8903	0.9233	1.051	0.9775	0.9477
Mean adult length	measures	402	385	352	165	191	156	474	352	157	178	153

Ratio is to mean adult lengths that are from Lu et al. (2020).

Table S3. Measure and ratio of skull of living black rhinoceros (mm). Related to Figure 1 and 4.

development stage	basal length		ratio	mendible length		ratio
	Mean	Range		Mean	Range	
1	221	213-229	0.4011	192		0.4174
2			0	248		0.5391
3	317		0.5753	274	266-280	0.5957
4	380		0.6897	316	307-323	0.687
5	435		0.7895	354	336-378	0.7696
6	465		0.8439	380	350-406	0.8261
7	477	428-525	0.8657	403	359-430	0.8761
8	511		0.9274	433	409-453	0.9413
9	503	476-530	0.9129	436	404-464	0.9478
10	516	493-534	0.9365	441	418-485	0.9587
11 (adult)	529	520-544	0.9601	450	425-478	0.9783
12	532	510-552	1	450	409-476	0.9783
13	536	517-560	0.971	461	425-484	1.0066
14	549	504-580	0.9946	454	423-484	0.9913
15	550	522-581	0.9964	458	427-487	1
16	548	504-585	0.9928	458	426-516	
17	550	517-582	0.9964	463	442-482	
18	557	536-598	1.0091	464	434-490	
19	575	545-618	1.0417	469	448-502	
20	567	546-592	1.0272	464	444-496	

Ratio is to mean adult length. All data are from Goddard, 1970.

Table S4. Measure and ratio of skull of living white rhinoceros (mm). Related to Figure 1 and 4.

	greatest length	ratio	basal length	ratio	condyle-nasal	ratio	upper tooth row	ratio	lower tooth row	ratio
1										
2										
3	304		326	0.47	302	0.47	125	0.46	125	0.47
4		0.41		0		0	160	0.59		0
5	480	0	530	0.76	498	0.77	202	0.75	205	0.76
6	565	0.64	590	0.85	533	0.82	203	0.75	199	0.74
7	637	0.75	630	0.91	593	0.92	197	0.73	202	0.75
8	674	0.85	670	0.96	588	0.91	230	0.85	231	0.86
9	657	0.9		0	593	0.92	228	0.84		0
10	695	0.88	663	0.95	611	0.94	245	0.91	250	0.93
11 adult	733	0.93	689	0.99	618	0.96	244	0.9	263	0.98
12	740	0.98	695	1	621	0.96	274	1.01	272	1.01
13	740	0.99	679	0.98	623	0.96	247	0.91	275	1.03
14	756	0.99	716	1.03	647	1	270	1	268	1
15	767	1.01								

Ratio is to mean adult length. All data are from Hillman-Smith, 1986.

Table S5. Biological traits of living rhinoceroses. Related to Figure 5.

Items	*C. simum*	*R. unicornis*	*D. bicornis*	*R. sondaicus*	*D. sumatrensis*
gestation	481-550 days	459-494 days	419-476 days	?	473-480 days
birth weight	48-60 kg	40-60 kg	23-48 kg	?	23-33 kg
weaning	1-2 years	1-2 years	1-2 years	1-2 years	1-2 years
sex mature (female)	3 years	3 years	4 years	3-4 years	4 years
first birth (female)	4-5 years	4-5 years	4-5 years	?	5 years
birth interval (minimum)	2-3 years	2-3 years	2-3 years	?	3-4 years
skeleton mature	10-12 years	6-10 years	8-10 years	?	10 years
body weight	1800-3600kg	1600kg-2400kg	886-1400 kg	900-1500 kg	600-800kg,
life span	35-50 years	40 years	20-34 years	30-40 years in wild	25-40 years
max span	57 years	47 years	57 years	?	47 years

Data of *Ceratotherium simum* (Groves, 1972; Hillman-Smith, 1986); *Rhinoceros unicornis* (Laurie et al., 1983); *Diceros bicornis* (Hillman-Smith and Groves, 1994); *Rhinoceros sondaicus* (Groves and Leslie, 2011); *Dicerorhinus sumatrensis* (Plair et al., 2012).

Table S6. Life span (year) estimation of living rhinoceroses and *Plesiaceratherium* based on body weight (kg). Related to Figure 5.

equations for life span estimation	*C. simum*	*D. bicornis*	*R. unicornis*	*R. sondaicus*	*D. sumatrensis*	*P. gracile*
Weight	2286	996	1844	1750	1046	1198
y=630W0.17 (Blueweiss, 1978)	21	18	20	20	18	19
y=4.11W0.162 (Magalhães et al., 2007)	45	40	44	43	40	41
y=3.34W0.193 (maximum, Magalhães et al., 2007)	56	48	54	54	48	50
y=2.66W0.22 (Western, 1979)	67	56	64	63	56	58

Body weight data of *Ceratotherium simum* (Groves, 1972; Hillman-Smith, 1986); *Rhinoceros unicornis* (Laurie et al., 1983); *Diceros bicornis* (Hillman-Smith and Groves, 1994); *Rhinoceros sondaicus* (Groves and Leslie, 2011); *Dicerorhinus sumatrensis* (Plair et al., 2012); *Plesiaceratherium gracile* (Lu et al. 2021).

Table S7. Sex maturity (year) estimation of living rhinoceroses and *Plesiaceratherium* based on body weight (kg). Related to Figure 5.

	C. simum	*D. bicornis*	*R. unicornis*	*R. sondaicus*	*D. sumatrensis*	*P. gracile*
y=0.214W0.263 (Magalhães et al., 2007)	6	5	6	6	5	5

Body weight data of *Ceratotherium simum* (Groves, 1972; Hillman-Smith, 1986); *Rhinoceros unicornis* (Laurie et al., 1983); *Diceros bicornis* (Hillman-Smith and Groves, 1994); *Rhinoceros sondaicus* (Groves and Leslie, 2011); *Dicerorhinus sumatrensis* (Plair et al., 2012); *Plesiaceratherium gracile* (Lu et al. 2021).

Table S8. Skull measure of two living African rhinoceroses and two fossil rhinoceroses. Related to Figure 5.

Dental development stage	*Diceros bicornis*	*Ceratotherium simum*	*Chilotherium simum*	*Plesiaceratherium gracile*
1	221			
2				
3	317	304		
4	380			407
5	435	480	363	
6	465	565		485
7	477	637	436	
8	511	674	474	572
9	503	657		
10	516	695		
11	529	733		
12	532	740	500	636.5
13	536	740	528	624.25
14	549	756	483	625.25
15	550	767		644
16	548			666
17	550			
18	557			
19	575			
20	567			

Data of *Ceratotherium simum* from Hillman-Smith, 1986; *Diceros bicornis* from Goddard, 1970; *Chilotherium simum* from Deng, 2001; *Plesiaceratherium gracile* from Lu et al., 2021.

Table S9. Skull length of ungulates in this study through the Paleogene to the Quaternary. Related to Figure 5.

species	references	age	geography	skull-length
Tetraclaenodon puercensis	Kondrashov et al., 2012	Early Paleocene	North American	114
Diacodexis metsiacus	Rose, 1982	Early Eocene	Asia-Eurpe	59
Cambaytherium thewissi	Rose, 2019	Early Eocene	Asia	163
Radinsky yupingae	Holbrook, 2014	Late Paleocene	Asia	89
Pliolophus vulpiceps	Owen, 1858	Early Eocene	Europe	127
Hyracotherium vasacciense	Kitts, 1956	Early Eocene	North American-Europe	135
Eurohippus messelensis	Franzen and Habersetzer, 2017	Middle Eocene	Europe	143
Orohippus osbornianus	Granger, 1908	Middle Eocene	North American	140
Eohippus venticolus	Granger, 1908	Middle Eocene	North American	138
Equus caballus	Bennett and Hoffmann, 1999	Holocene	Asia	527
Lambdotherium popoagicum	Lucas and Holbrook, 2004	Early Eocene	North American	186
Embolotherium andrewsi	Mihlbachler, 2008	Middle Eocene (Duchesnean)	North American	728
Litolophus gobiensis	Bai et al., 2010	Early Eocene	Asia	182
Lophiaspis maurettei	Vautrin et al., 2021	Early Eocene	Europe	230
Moropus petersoni	Peterson, 1907	Late Oligo-Early Mio	North American-Europe	355
Ancylotherium pentelicum	Geraads et al., 2007	Miocene	Europe	668
Homogalax protapirinus	Holbrook et al., 2004	Early Eocene	North American	165
Isectolophus latidens	Holbrook et al., 2004	Early Eocene	North American	170
Heptodon	Radinsky, 1965	Early Eocene		>195

posticus				
Eoletes gracilis	Lucas et al., 1997	Early Eocene	Kazakhstan	126
Irenolophus qii	Bai et al., 2019	Early Eocene		241
Colodon occidentalis	Colbert, 2004	Early Oligo	North American	230
Tapirus terrestris	Padilla and Dowler, 1994	Holocene	South American	335
Hyrachyus modestus	Bai et al., 2017	Middle Eocene	North American	228
Rostriamynodon grangeri	William and Manning, 1986	Late Eocene	North American-Asia	560
Amynodon advenus	Wall, 1982	Late Eocene	North American	487
Triplopus obliquidense	Peterson, 1919	Late Eocene	North American	165
Hyracodon nebraskensis	Scott, 1941	Early Oligocene	North America	291
Juxia sharamurense	Qiu and Wang, 2007	Middle Eocene	Asia	595
Paraceratherium lepidum	Qiu and Wang, 2007	Late Oligocene	Asia	1325
Teletaceras radinskyi	Hanson, 1989	Middle Eocene	North American	343
Trigonias osborni	Prothero, 2005	Late Eocene	North American	515
Aprotodon lanzhouensis	Deng, 2006	Late Oligocene-Early Miocene	Asia	625
Diaceratherium shanwangensis	Lu et al., 2021	Late Oligocene-Early Miocene	Europe-Asia	526
Plesiaceratherium	Lu et al., 2020	Early Miocene	Europe-Asia	648
Coelodonta antiquitatis	Deng et al., 2011	Pleistocene	Europe-Asia	780
Rhinoceros unicornis	IVPP OV 1383	Holocene	Asia	675
Ceratotherium simum	IVPP OV 2629	Holocene	Africa	797
